# Decreased Physical Activity as an Early Digital Biomarker in Huntington's Disease: A One‐Year Observational Study

**DOI:** 10.1002/brb3.71149

**Published:** 2026-02-05

**Authors:** Lucía Simón‐Vicente, Sara Calvo, Natividad Mariscal, Ignacio Muñoz‐Siscart, Dolores Diaz‐Piñeiro, Jéssica Rivadeneyra, Esther Cubo

**Affiliations:** ^1^ Research Unit Burgos University Hospital Burgos Spain; ^2^ Neurology Department Burgos University Hospital Burgos Spain; ^3^ Psychiatry Department Burgos University Hospital Burgos Spain; ^4^ Health Science Department University of Burgos Burgos Spain

**Keywords:** activity monitors, Huntington's disease, sarcopenia, wearables, physical activity

## Abstract

**Introduction:**

Huntington's disease (HD) is a neurodegenerative disorder characterised by motor dysfunction, cognitive impairment, and psychiatric disturbances. This study analyzed the relationship between clinical characteristics, sarcopenia, and physical activity (PA) levels in HD patients.

**Methods:**

A 1‐year observational study was conducted with symptomatic, ambulatory HD patients, assessed at baseline and after 12 months. PA was monitored using a Fitbit Charge 4 activity tracker, and sarcopenia was determined through assessments of muscle strength, quantity, and physical performance. Participants were classified into two clusters based on age, motor (Unified HD Rating Scale), and cognitive function (Mini‐Mental State Examination).

**Results:**

We included 33 subjects with HD, mean age 53 (40–60) years, 45.5% males, median TFC 9.5 (7–13). At baseline, Cluster 1 had better motor function, functional capacity, and less apathy with a positive trend for higher PA, compared with Cluster 2, which had a negative trend for decreased PA over time (*p* = 0.006). After 1 year, Cluster 1 showed a decrease in PA (*p* = 0.035), similar to Cluster 2. At baseline, 53% of participants in Cluster 2 presented probable or confirmed sarcopenia, compared with 13% in Cluster 1. Significant differences (*p* < 0.05) were observed in muscle strength, bioelectrical impedance analysis (BIA), and SPPB scores, with higher values in Cluster 1.

**Conclusions:**

These preliminary findings suggest that a reduction in PA using wearable technology may be a potential early indicator of functional changes in HD. Identifying when PA reduction begins can help determine the timing of interventions aimed at delaying disease progression.

## Graphical abstract

Physical Activity: Early Biomarker in Huntington's Disease.

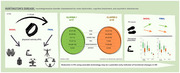

## 1 Introduction

Huntington's disease (HD) is an autosomal dominant neurodegenerative disease caused by a mutation in the HTT gene on chromosome four, with abnormal repetition of the cytosine–adenine–guanine (CAG) nucleotide. Clinically, HD is characterised by motor dysfunction, predominantly chorea, cognitive impairment, and psychiatric disturbances such as apathy or depression (Kumar et al. 2020).

As the disease progresses, symptoms, including skeletal muscle wasting, weight loss, sarcopenia, and cachexia, become relevant, leading to decreased quality of life, increased comorbidity, and risk of mortality (Di Renzo et al. 2021; Ghosh and Tabrizi 2018). Sarcopenia involves progressive loss of skeletal muscle mass and strength, with a risk of adverse outcomes such as physical disability, poor quality of life, and death (Cruz‐Jentoft et al. 2019). Furthermore, this disorder causes muscle weakness, particularly in lower limbs, affecting walking, standing, climbing stairs, or transferring from a chair to a standing position, thereby reducing the patient's ability to perform basic activities of daily living (Lan et al. 2024; Nakahara et al. 2021). Frailty and sarcopenia occur more frequently in patients with neurodegenerative disorders and are associated with a more adverse disease course (Peball et al. 2019).

Despite advances in symptomatic pharmacological treatments for controlling involuntary movements, no current therapy modifies the HD course. Nonpharmacological interventions, particularly exercise and physical activity (PA), have shown beneficial effects on cognitive and motor symptoms and are associated with better functional outcomes and prolonged independence (Vizzi et al. 2020). Conversely, reduced PA has been linked to greater functional dependence and earlier institutionalisation in HD patients (Wheelock et al. 2003).

Recent technological advances have enabled continuous monitoring of PA using wearable sensors (e.g., Fitbit, ActiGraph, and Garmin), providing objective and longitudinal data on mobility in real‐world environments (Ward et al. 2005). These devices assess sleep‐wake patterns, heart rate, cardio fitness level, and energy expenditure, and are used by researchers to collect PA data over time. Step count has become a widely accepted outcome measure in PA research (Evenson et al. 2015; Rayward et al. 2021; Saygin et al. 2022), offering an accessible and quantifiable marker of daily activity in several neurological disorders. Unlike single‐time assessments, wearables enable real‐time, ecologically valid monitoring of walking behavior, reflecting motor and motivational changes over time. Monitoring and understanding whether PA is an early digital biomarker in people with HD can inform strategies to mitigate inactivity and may improve symptom management, enhancing overall health, quality of life, and prevention of functional decline (Dasmahapatra et al. 2018).

We hypothesized that declining PA, quantified by daily step count, may serve as a digital behavioral biomarker of early functional deterioration in HD. While traditional biomarkers (e.g., neuroimaging and cerebrospinal fluid markers) are typically biological and proximal to pathology, digital biomarkers are objective, quantifiable physiological and behavioral data from digital devices that indicate disease processes (Coravos et al. 2019; Lipsmeier et al. 2018). Behavioral digital biomarkers, like PA levels, can capture subtle changes in functioning that precede clinical milestones, particularly in prodromal neurodegenerative diseases (Janssen Daalen et al. 2024).

Since motor and nonmotor features (e.g., apathy, cognition, and medication side effects) can influence PA, changes in step count might reflect multiple factors in early neurodegeneration pathophysiology. Therefore, this study aimed to analyze the longitudinal relationship between clinical features, sarcopenia, and PA levels in HD patients over one year. We propose that a decline in PA, reflected by step count, precedes or parallels clinical deterioration in HD and may represent an early, ecologically valid digital biomarker of functional decline in HD patients.

## 2 Materials and Methods

### 2.1 Design

This exploratory, longitudinal, observational study was conducted at the Neurology Department of Burgos University Hospital, Spain. The design and methodology followed the guidelines for observational studies (STROBE) (Von Elm et al. 2007).

### 2.2 Participants

We recruited a convenience sample of symptomatic, ambulatory individuals diagnosed with HD with a confirmed genetic mutation of > 36 CAG repeats in the HTT gene. Participants were evaluated by neurologists with extensive experience in HD. Symptomatic HD participants were defined with a score greater than 4 on the motor subdomain of the Unified Huntington's Disease Rating Scale (UHDRS)(Kieburtz 1996), and a diagnostic confidence level (DCL) of 4, able to walk with minimal support. Participants diagnosed with diabetes mellitus, thyroid disturbances, active cancer, other neurodegenerative conditions, or cardiac, pulmonary, or skeletal–muscular diseases were excluded. Individuals who were pregnant, breastfeeding, or taking medications that could affect metabolism/endocrine function were also excluded. This study was conducted following the Good Clinical Practice standards (Association 2013) involving humans and was approved by the Institutional Review Board (Complejo Universitario Burgos and Soria, Certificate number: CEIM‐2429. All participants provided informed consent by signing a consent form before participation.

### 2.3 Study Protocol

The participants were assessed at baseline and 12 months later. Baseline assessments included an interview to gather sociodemographic and clinical information.

We collected basic information using a standard questionnaire, including age, sex, education level, marital status, and smoking history, and measured height and weight on‐site to calculate the body mass index (BMI; weight/height^2^).

All HD participants were evaluated by certified movement disorder neurologists at baseline and at the end of the study using the UHDRS battery (Kieburtz 1996), including the motor subscale (UHDRS‐TMS) (Kieburtz 1996), with high scores denoting greater impairment. The Mini‐Mental State Examination (MMSE) and the cognitive test battery from the UHDRS (“cogscore”; Stroop color naming, word reading, and interference tests; the phonemic verbal fluency test with the letters F, A, and S; and the symbol digit modalities test [SDMT]) were used to evaluate cognitive function (Ringkøbing et al. 2020). The 12‐item short form‐12 health survey (SF‐12) was used to assess health‐related quality of life (Shah and Brown 2020), and functional capacity using the total functional capacity (TFC)(Marder et al. 2000). Finally, clinical disease progression was calculated using the composite unified Huntington's disease rating scale (cUHDRS)(Estevez‐Fraga et al. 2021), a multidomain measure encompassing motor, functional, and cognitive scales independently associated with HD severity. The four subscales from which the score was derived were the TFC, UHDRS‐TMS, symbol‐digit modality test (SDMT) (Parmenter et al. 2007), and Stroop word reading (SWR) test (Scarpina and Tagini 2017; Aylward et al. 2013; Poudel et al. 2014).

Subsequently, a series of assessments was conducted to evaluate muscle strength, quantity, and physical performance. Participants were instructed to wear an activity tracker (Fitbit Charge 4) on their nondominant wrist for 12 months, and therapists reviewed their activity levels using the Fitabase web‐based program.

### 2.4 Assessment of Sarcopenia

Sarcopenia was defined according to the 2019 consensus update issued by the European Working Group on Sarcopenia in Older People (EWGSOP2)(Cruz‐Jentoft et al. 2019).

#### 2.4.1 Muscle Strength

Handgrip strength was evaluated using a Jamar dynamometer. Participants were seated with shoulders adducted in a neutral position, forearms neutral, elbows flexed to 90°, and wrists slightly extended (Hamilton et al. 1992). Participants were asked to squeeze the dynamometer with maximal effort for three trials, with mean scores recorded for each upper extremity. Low muscle strength (LMS) was defined as *<* 27 kg in men and *<* 16 kg in women (Cruz‐Jentoft et al. 2019).

#### 2.4.2 Muscle Quantity

Muscle quantity was measured using bioelectrical impedance analysis (BIA; body composition analyzer Seca mBCA 525 [Hamburg, Germany]), with eight electrodes. The impedance was measured with a current of 100 µA at frequencies of 1, 2, 5, 10, 20, 50,100, 200, and 500 kHz and an impedance measuring range of 10 to 1000 Ω.

BIA measures the electrical properties of body tissue based on resistance from total water across the body to a small alternating current. Fat mass is considered a nonconductor of electric charge and is equal to the difference between body weight and fat‐free mass (FFM). FFM is considered the conducting volume that helps pass electric current owing to the conductivity of electrolytes dissolved in body water. BIA uses three methods: single frequency, multiple frequencies (mfBIA), and bioimpedance spectroscopy (Fosbøl and Zerahn 2015; Khalil et al. 2014).

#### 2.4.3 Physical Performance

The short physical performance battery (SPPB) evaluates physical function and functional strength through three different tests: standing balance test, walking, and chair sit‐to‐stand. For the standing balance test, participants were required to stand unassisted in side‐by‐side, semitandem, and tandem positions. Participants were then asked to walk a 4 m distance from a standing start. Finally, they were asked to stand up from a chair as quickly as possible without using their arms five times. The total score ranged from 0 to 12, with higher scores indicating greater lower extremity functional strength (Guralnik et al. 1994).

### 2.5 Assessment of Daily Step Count

The Fitbit Charge 4, is a wrist‐worn activity monitor (3.58 × 2.27 × 1.25 cm and weighs 20 g) that uses accelerometer, gyroscope/altimeter, and heart rate sensor data to estimate daily PA metrics (such as step counts in minutes, hours, and days granularity, number of floors climbed, and intensity of activity performed), sleep (such as total minutes asleep and time spent in different sleep stages), and heart rate. The scores were displayed and uploaded to the Fitbit website, where an overview of PA was presented. Daily step counts were extracted from the accelerometer to obtain the average daily step count. Raw data were exported to CSV and then converted into an Excel file for data interpretation.

The primary measure of PA quantity was average daily step count, defined as total steps per day averaged over device wear days (Graphical Abstract).

Adherence to wearing the FB was assessed based on the following criteria: (1) days with fewer than 100 steps were invalid; (2) minimum of 11 valid days with step data required in first and last 28‐day periods; (3) more than 20 valid data days were required to perform the 3‐month linear regression analysis; and (4) for patients who did not have data during the first or last 4 consecutive weeks of the recording period, the first or last week with available data within the initial or final month was considered the starting or ending week, respectively.

### 2.6 Statistical Analysis

Descriptive statistics for participants and main outcomes are presented as the mean and standard deviation (SD) for continuous variables, and median, and 25th–75th percentiles for nonnormally distributed or ordinal data. Normality of the variables was evaluated using the Shapiro–Wilk test. Frequency distribution and percentages describe categorical variables.

To calculate the number of daily steps recorded by the smartwatch, the trimmed mean α (with *α* = 0.2) of the first 28 days (4 weeks) of Fitbit data was used to match the time at which the baseline and final assessments were performed. We used 3 months of data to calculate the regression line coefficient. Spearman's correlation coefficients examined associations between PA estimates (mean, median step count) and clinical variables, with moderate (*r* = 0.40–0.75) and high correlation (*r* > 0.75) (Fleiss et al. 1999).

In addition, patients were classified into clusters according to age, UHDRS, and MMSE scores at baseline. For this purpose, Ward's hierarchical agglomerative and nonhierarchical k‐means procedures were applied together. Based on the squared Gower distance, Ward's method facilitated the identification of the optimal number of clusters and outlier cases. We used the Chi‐Square test (or Fisher's test) for qualitative variables and the nonparametric Mann–Whitney *U*‐test for quantitative variables to analyze differences between the study variables in the different clusters. Finally, we used the Wilcoxon and McNemar repeated‐measures tests to analyze differences between clusters over time. *p*‐values < 0.05 were considered statistically significant. The Benjamini–Hochberg correction was applied to analyses involving multiple comparisons.

We used IBM‐SPSS version 29.0 software (IBM SPSS Statistics for Windows, IBM, Armonk, NY, USA) and R software for analysis.

## 3 Results

We included 37 subjects with HD; however, we excluded four subjects due to missing data for heart rate recordings from the Fitbit. Therefore, 33 participants were included in this study, with a median age of 53 (40–60) years (range), and 45.5% males and 54.5% females. The demographic characteristics of the participants are shown in Table 1.

**TABLE 1:** Sociodemographic and clinical characteristics of the sample.

Total (*n* = 33)	—
Age (years), median (IQR)	53 (40–60)
Sex	—
Male, *n* (%)	15 (45.5)
Female, *n* (%)	18 (54.5)
CAG repeat length, median (IQR)	43 (41–45)
Body mass index, median (IQR)	25.55 (23–28)
UHDRS‐TMS, median (IQR)	21.5 (4–43)
TFC, median (IQR)	9.5 (7–13)
cUHDRS, median (IQR)	10.92 (5–16)
SF‐12, median (IQR)	—
Physical component	50.82 (47–55)
Mental component	54.69 (51–58)
PBA, median (IQR)	—
Depression	1 (0–10)
Irritability	0 (0–2)
Phsycosis	0
Apathy	0 (0–4)
Executive functions	0 (0–3)
MMSE, median (IQR)	29 (28–30)
UHDRS cogscore, median (IQR)	199 (127–285)
Fatigue self‐assessment, median (IQR)	—
Physical	2 (0–5)
Mental	3 (2–7)
Energy (kcal), median (IQR)	2776.08 (2328.3–3222)

Abbreviations: UHDRS‐TMS, Total Motor Score; TFC, Total Functional Capacity; cUHDRS, composite Unified Huntington's Disease Rating Scale; PBA, Problems Behavioral Assessment; IQR, Interquartile Range; MMSE, Mini‐Mental State Examination; SF‐12, Short‐form health survey 12; UHDRS cogscore, Unified Huntington's Disease Rating Scale Cognitive Score.

### 3.1 Associations Between Physical Activity and Clinical Status

We found a moderate positive correlation between MMSE scores (*r* = 0.45, *p* = 0.016) and daily step counts. No statistically significant correlations or differences were observed with the other clinical scales.

### 3.2 Cluster Analysis

Two distinct clusters were identified in this study. The characteristics of the two clusters are presented in Table 2. Cluster 1 comprised 51.51% (*n* = 17) of participants, and it was characterized by significantly better motor function, functional capacity, physical health‐related quality of life, and less apathy compared with Cluster 2. Likewise, Cluster 2 comprised 48.48% (*n* = 16) of the participants and was characterised by older age and greater disease progression than Cluster 1.

**TABLE 2:** Demographic and clinical characteristics of the different clusters of Huntington's disease.

	BASAL			FINAL		
Clinical characteristics	Cluster 1 (*N* = 17)	Cluster 2 (*N* = 16)	*p*‐value	Cluster 1 (*N* = 17)	Cluster 2 (*N* = 11)	*p*‐value
Sex						
Male, *n* (%)	8 (47.1%)	7 (43.8%)	0.934	8 (47.1%)	5 (45.5%)	0.934
Female, *n* (%)	9 (52.9%)	9 (56.3%)		9 (52.9%)	6 (4.5%)	
CAG repeat length, median (IQR)	45 (41–46)	41.5 (41–43)	0.187	45 (41–46)	41.5 (41–43)	0.208
Age (years), median (IQR)	40 (35–45)	60 (58–66)	**<0.001**	41 (36–46)	61 (59–67)	**<0.001**
UHDRS‐TMS, median (IQR)	5 (1–11)	44 (35–55)	**<0.001**	7 (2–17)	40 (22–52)	**<0.001**
TFC, median (IQR)	13 (12–13)	7 (5–9)	**<0.001**	13 (11–13)	7 (6–9)	**<0.001**
cUHDRS, median (IQR)	16.17 (14–18)	6.32 (4–9)	**<0.001**	14.93 (12–18)	6.65 (4–9)	**<0.001**
Independence scale, median (IQR)	100 (95–100)	75 (63–80)	**<0.001**	100 (85–100)	75 (65–80)	**0.003**
Function, median (IQR)	25 (25–25)	17 (11–22)	**<0.001**	25 (24–25)	19.5 (15–21)	**<0.001**
MMSE, median (IQR)	29 (29–30)	29 (26–30)	0.464	29 (29–30)	28 (28–29)	0.341
UHDRS cogscore, median (IQR)	282 (235–342)	154 (115–198)	0.003	285 (214‐350)	160 (132–194)	0.003
PBA, median (IQR)						
Depression	1 (0–9)	2 (0–10)	0.915	1 (0–5)	5 (0–8)	0.791
Irritability	0 (0–1)	1 (0–4)	0.466	0 (0–2)	0 (0–4)	0.649
Phsycosis	0 (0–0)	0 (0–0)	0.999	0 (0–0)	0 (0–0)	0.860
Apathy	0 (0–0)	4 (1–6)	**0.003**	0 (0–1)	4 (2–8)	**0.008**
Executive Functions	0 (0–5)	0 (0–2)	0.466	0 (0–4)	0 (0–3)	0.860
Total	2 (0–16)	7 (1–21)	0.466	6 (1–13)	11 (7–20)	0.369
SF‐12, median (IQR)						
Physical component	55.05 (51–56)	48.39 (43–51)	**0.005**	53.83 (45–56)	46.29 (40–54)	0.369
Mental component	51.5(51–57)	55.45 (52–61)	0.340	53.85 (40–57)	45.89 (33–56)	0.649
Fatigue self‐assessment, median (IQR)						
Physical	2 (0–5)	2.5 (1–5)	0.915	3.5 (1–6)	4 (3–6)	0.649
Mental	4 (2–7)	3 (2–7)	0.915	3 (1–8)	5 (3–8)	0.649
Body mass index, median (IQR)	25.78 (23–35)	24.5 (23–27)	0.464	25.71 (23–34)	25.32 (23–28)	0.649
Energy (kcal), median (IQR)	2905.9438 (2.312–3.247)	2769.3 (2.327–3.207)	0.944	3210.88 (2341–3470)	2783.85 (2232–3247)	0.525

*Note*: *p*‐values adjusted using the Benjamini–Hochberg method. Statistical tests: χ^2^ for dichotomous variables and Mann–Whitney *U* test for continuous variables.

Abbreviations: UHDRS‐TMS, total motor score; TFC, Total Functional Capacity; cUHDRS, composite Unified Huntington's Disease Rating Scale; PBA, Problems Behavioral Assessment; IQR, Interquartile Range; MMSE, Mini‐Mental State Examination; SF‐12, Short‐form health survey 12; UHDRS cogscore, Unified Huntington's Disease Rating Scale Cognitive Score.

**FIGURE 1:**
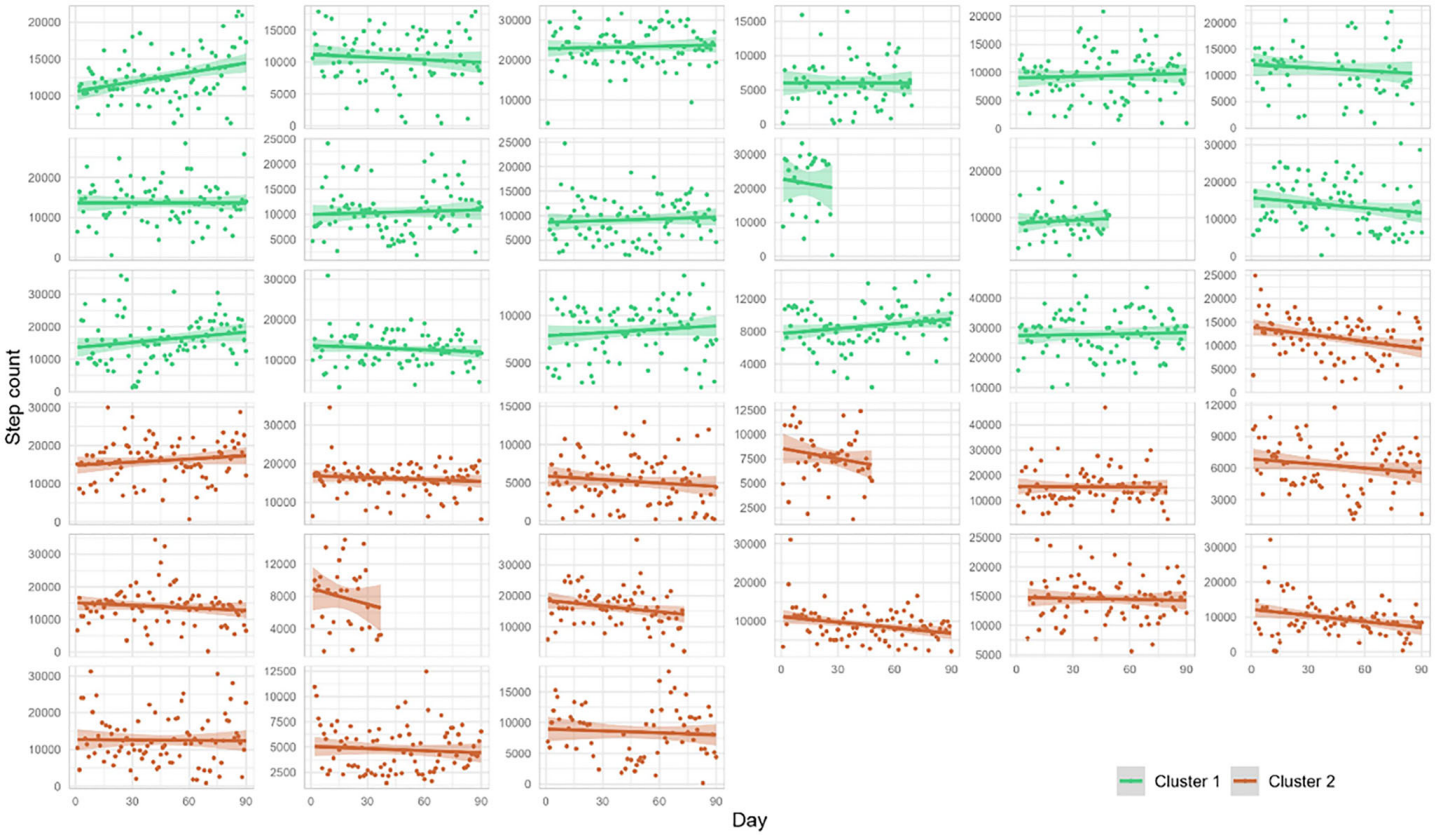
Steps taken by each patient during the first 3 months of Fitbit recording, along with their trend line.

**FIGURE 2:**
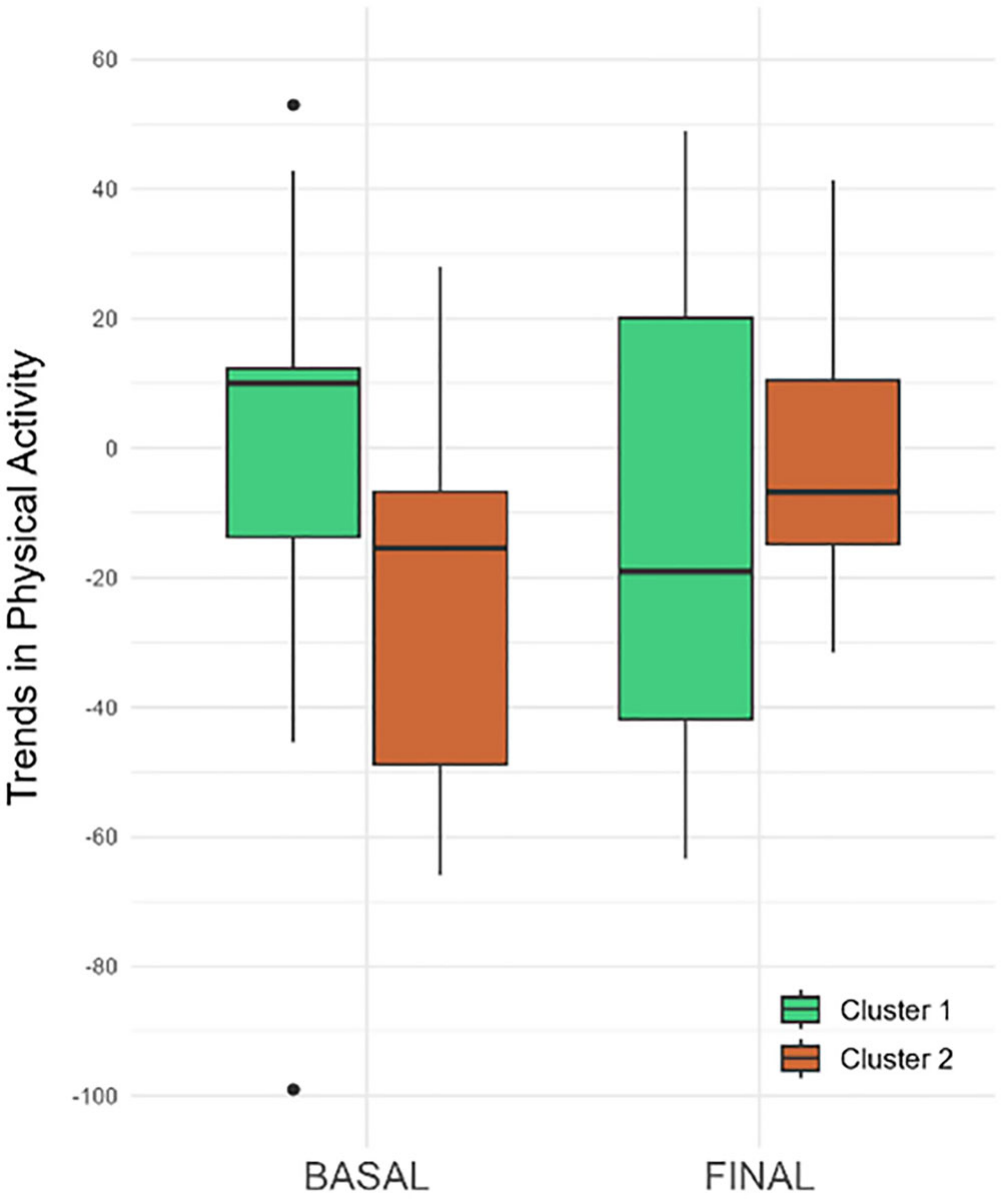
Box plot of the physical activity trend in both clusters at the beginning and end of the study.

Regarding the level of PA performed, both Clusters were similar in terms of daily steps taken (11.467 [8.771–15.012]) versus (11.490 [8.064–14.106]; *p* = 0.606; Figure 1). In contrast, significant differences were found in PA trends over time, with Cluster 1 showing a greater and positive median trend for higher PA, while Cluster 2 had a negative trend for PA (*p* = 0.006; Figure 2).

**FIGURE 3:**
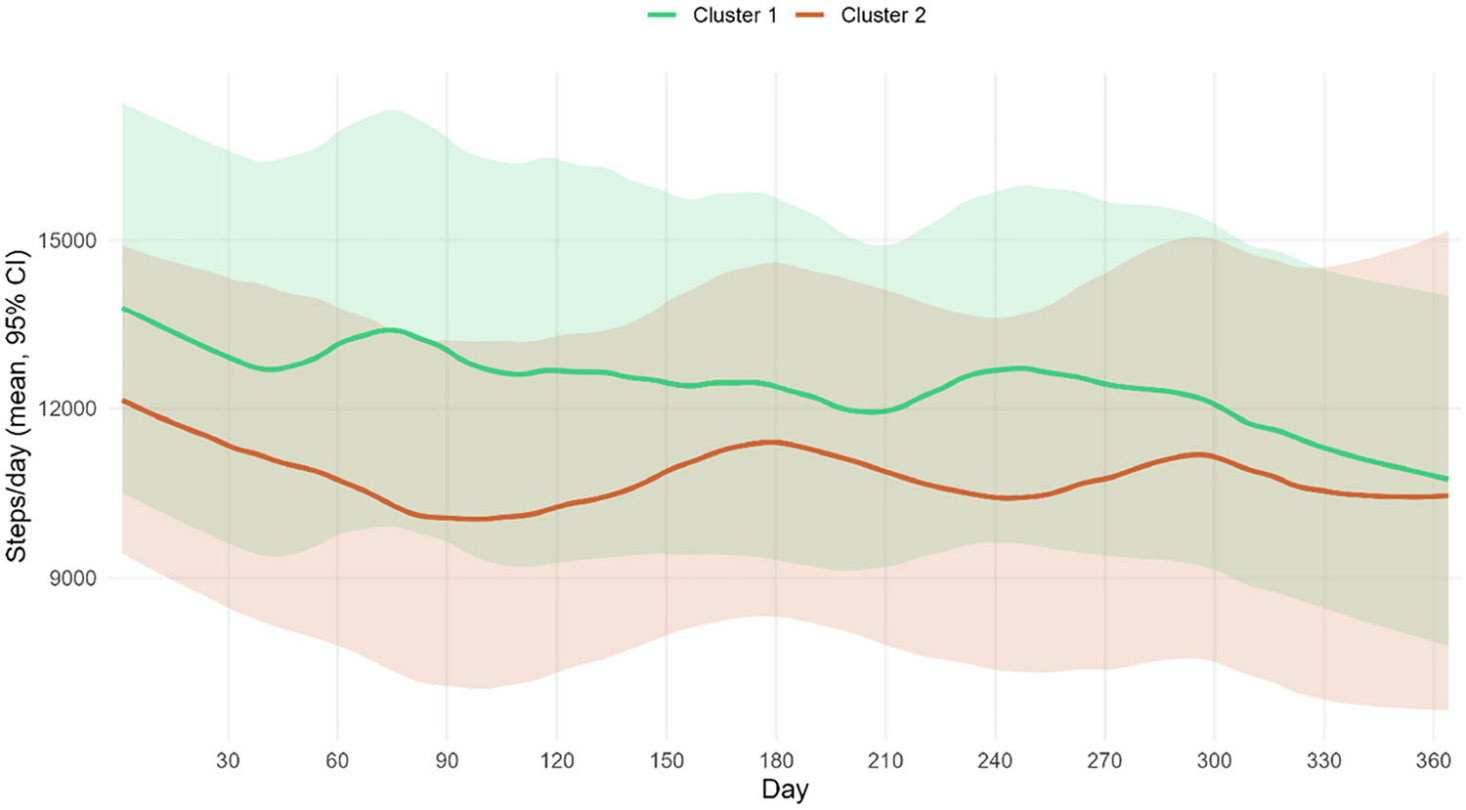
Average daily step trajectories by cluster.

After 1 year, no clinical differences over time were observed within each cluster. Regarding PA, patients in Cluster 1 showed a decrease in the number of steps over time (final steps: 9.737 [7.885; 13.206]); in contrast, no statistically significant differences were found in Cluster 2 (final steps: 10.465 [6.329; 15.683]; Figures 1 and 3). Interestingly, the trend of decreasing PA over the year in Cluster 1 aligned with that of Cluster 2 (Figures 2 and 3).

Regarding sarcopenia, at baseline, 13.3% of patients in Cluster 1 presented with probable sarcopenia; in Cluster 2, 40% had probable sarcopenia, 6.7% had confirmed sarcopenia, and 6.7% had severe sarcopenia. At the final assessment, 6.3% of patients in Cluster 1 had probable sarcopenia, and 6.3% had confirmed sarcopenia. In Cluster 2, at the final time point, 57.1% had probable sarcopenia and 14.3% had severe sarcopenia. Statistically significant differences were observed in the presence of sarcopenia between Clusters 1 and 2 at baseline (*p* = 0.028) and final assessment (*p* = 0.002).

Concerning sarcopenia parameters, no statistically significant differences were observed in Clusters 1 and 2 over the course of 1 year, although differences were found between the clusters (Table 3).

**TABLE 3:** Sarcopenia parameters of the different clusters of Huntington's disease.

			BASAL			FINAL		
			Cluster 1 (*n* = 17)	Cluster 2 (*n* = 16)	*p*‐value	Cluster 1 (*n* = 16)	Cluster 2 (*n* = 14)	*p*‐value
Muscle strength			31 (24–40)	19 (12–26)	**0.024**	25 (21–36)	18 (13–21)	**0.002**
	Sarcopenia, %	no	86.67	46.7	**0.028**	87.50	28.57	**0.002**
		yes	13.33	53.30		12.50	71.43	
Muscle quantity (BIA)			25 (19–31)	18 (17–24)	**0.028**	27 (20–30)	18 (16–24)	0.055
	Sarcopenia, %	no	100.00	81.25	0.103	93.33	85.71	0.598
		yes	0.00	18.75		6.67	14.29	
SPPB			12 (10–12)	8 (6–9)	**<0.001**	12 (11–12)	8 (6–9)	**<0.001**
	Sarcopenia, %	no	93.33	46.67	**0.028**	100.00	23.08	**<0.001**
		yes	6.67	53.33		0.00	76.92	

*Note*: χ^2^ for dichotomous variables and Mann–Whitney *U* test for continuous variables.

Abbreviations: BIA, Bioelectrical Impedance Analysis; SPPB, Short Physical Performance Battery.

## 4 Discussion

This study aimed to analyze the relationship between clinical characteristics, sarcopenia, and PA levels in patients with HD over a year. We found that patients with better functional status, motor performance, and lower psychiatric symptoms (Cluster 1) were more physically active at baseline than Cluster 2. Notably, Cluster 1, comprising early manifest stage patients, showed significant PA decline over time, despite initially preserved clinical function.

These findings align with evidence suggesting that early changes in motor control and mobility precede clinical deterioration. For instance, Reyes et al. (2018) identified impairments in postural control in premanifest and manifest HD patients, suggesting balance alterations as early markers of disease progression. Similarly, Alzakerin et al. (2019) reported altered stride pattern stability in HD, although there was no clear distinction between the premanifest and manifest stages. Our observation of early PA decline in the mildly affected group provides evidence that objectively measured free‐living PA may reflect subtle motor dysfunction in HD. Preclinical studies show voluntary exercise can delay disease onset and attenuate neuropathological changes in HD mouse models (Pang et al. 2006; Van Dellen et al. 2008). These findings provide a biological basis for the hypothesis that PA decline in the early stages of the disease may not only reflect functional impairment but also relate to the underlying neurodegenerative mechanisms.

Similar approaches have been explored in other neurodegenerative conditions, particularly Parkinson's disease (PD), where PA and gait declines precede clinical diagnosis. For example, longitudinal studies in PD found reduced step count and lower gait speed years before motor symptoms manifest, supporting the use of wearable‐based monitoring for early disease detection (Del Din et al. 2016; Sotirakis et al. 2023). Furthermore, a recent study by Bianchini et al. (2025) demonstrated that in mild‐to‐moderate PD, changes of 581–1592 steps/day corresponded to clinically important mobility differences, supporting that step counts may serve as early HD indicators. These findings add weight to the notion that step counts derived from everyday wearables can capture meaningful changes in ambulatory activity, thus supporting our hypothesis that similar declines in step counts may serve as early indicators of HD.

Unlike findings in PD and Alzheimer's disease, where prodromal motor and nonmotor markers are well characterised (Berg 2008; Schneider et al. 2012), identification of early indicators in HD remains limited. Wang et al. (2024) found that increased light PA was causally associated with delayed age at onset (AAO) of HD, while sedentary behavior led to earlier AAO. Our findings align with this evidence and support the need to monitor PA trajectories over time, especially in at‐risk individuals or early disease stages.

Although exploratory and based on a small cohort, our results suggest that PA deterioration may begin before a major clinical decline, potentially offering a window for intervention. However, further research must determine whether PA decline reflects neurodegenerative processes, behavioral changes, or contextual factors, such as the environment or lifestyle.

Our findings suggest PA deterioration could begin earlier than previously recognised, during a window when neurodegenerative changes are detectable but before substantial clinical impairment emerges. Rather than indicating a definitive biomarker, early PA decline may represent a potentially informative and accessible signal for investigation, given the widespread availability of wearable PA devices in clinical and community settings (Willingham et al. 2024).

Moreover, exercise interventions in PD have demonstrated benefits not only in motor symptoms but also in nonmotor domains, such as mood and cognition, suggesting PA's neuroprotective role, associated with lower levels of depression and inflammatory markers, both of which are highly relevant given the prevalence of apathy, irritability, and neuroinflammation in HD (Ahlskog 2011; Goodwin et al. 2008; Guo and Le 2024). Moreover, regular PA has protective effects and may modulate neurodegenerative processes through both peripheral and central mechanisms (Sujkowski et al. 2022). These findings highlight the relevance of studying PA trajectories in HD, as similar mechanisms may be involved in early disease expression and progression. Although our study was not designed to assess causal pathways, these findings support the rationale for future studies to investigate whether increasing PA could have symptomatic and disease‐modifying effects in HD.

With respect to sarcopenia, our findings revealed clear differences between clusters in muscle strength, muscle quantity, and physical performance (SPPB), but no significant differences in body composition over one year. This suggests that while muscle mass may remain relatively stable in HD patients receiving standard care, strength loss could emerge earlier in advanced clinical stages. In contrast to the decline in PA, sarcopenia did not deteriorate early in our cohort and, therefore, should not be considered an early biomarker of neurodegeneration in HD. Notably, PA interventions have the potential to improve strength and function in HD; however, the long‐term effects of PA on sarcopenia in this population remain to be determined (Cabanas‐Valdés et al. 2022).

This study had limitations. First, the small sample size limited the detection of clinically relevant effects, reducing conclusion robustness. Due to the rarity of symptomatic HD and the challenges of year‐long wearable monitoring, recruitment was limited. Therefore, we did not perform a formal power analysis; the study was designed as an exploratory investigation to identify trends for future research. Second, while gait variability parameters are recognised as digital biomarkers in HD (Gaßner et al. 2020), Fitbit's accuracy for step count and activity intensity lacks formal validation in this population. Gait abnormalities, chorea, and other motor symptoms may lead to misestimation of free‐living PA using wrist‐worn devices. However, studies of Fitbit accuracy in PD, which involves gait slowness, freezing, and dyskinesias, show high correlation with criterion‐standard measures, suggesting that Fitbit can capture irregular steps reasonably well. Validation studies in HD are needed to establish metric accuracy. Third, we enrolled an ambulatory sample from an HD clinic, limiting generalisability. Fourth, although we captured behavioral symptoms with the PBA, residual confounding by behavioral, cognitive, and psychological disease‐related features (e.g., apathy, depression/anxiety, executive dysfunction) and nondisease‐related factors such as seasonal variation, and occupation status may influence PA independently of motor status. Future studies should incorporate multivariate analyses that explicitly adjust for behavioral domains (and other contextual factors such as age, sex, BMI, CAG repeat length, season, and occupational status) to better disentangle motor versus motivational contributions to PA. Finally, device‐derived metrics may be affected by gait abnormalities and hyperkinetic movements, requiring broader validation in HD.

Despite these limitations, our findings are innovative; to our knowledge, this is the first study to examine the progression of manifest and premanifest HD based on PA and body composition using free‐living wearable monitoring that more closely reflects everyday conditions.

## 5 Conclusion

In conclusion, the findings of this study provide preliminary insights into how activity‐tracking technology can be implemented in clinical settings to address the common barriers to exercise among individuals with HD. Our results contribute to the characterisation of PA in people with HD and may support future efforts to use wearable technology and digital biomarkers, potentially informing the design and timing of future intervention studies. However, further research involving larger HD cohorts is required to address this therapeutic challenge more comprehensively.

## Author Contributions

**Lucía Simón**: conceptualization, data curation, formal analysis, investigation, methodology, project administration, validation, visualization, writing – original draft preparation. **Sara Calvo**: conceptualization, formal analysis, investigation, methodology, visualization, writing – original draft preparation. **Natividad Mariscal**: data curation, investigation. **Ignacio Muñoz**: data curation, software, methodology, supervision. **Dolores Diaz**: conceptualization, validation, writing – review & editing. **Jéssica Rivadeneyra**: conceptualization, validation**. Esther Cubo**: conceptualization, methodology, investigation, writing – original draft preparation.

## Funding

The authors have nothing to report.

## Conflicts of Interest

The authors declare no conflicts of interest.

## Ethics Statement

The Institutional Review Board (Complejo Universitario Burgos and Soria) approved the study (certificate number: CEIM‐2429).

## Patient Consent Statement for Work with Human Subjects

All participants provided informed consent by signing the consent form before participating.

## Data Availability

The data that support the findings of this study are available on request from the corresponding author upon reasonable request.
